# Investigating the Morphology and Mechanics of Biogenic Hierarchical Materials at and below Micrometer Scale

**DOI:** 10.3390/nano12091549

**Published:** 2022-05-03

**Authors:** Mohammad Soleimani, Sten J. J. van den Broek, Rick R. M. Joosten, Laura S. van Hazendonk, Sai P. Maddala, Lambert C. A. van Breemen, Rolf A. T. M. van Benthem, Heiner Friedrich

**Affiliations:** 1 Laboratory of Physical Chemistry and Center for Multiscale Electron Microscopy, Department of Chemical Engineering and Chemistry, Eindhoven University of Technology, Groene Loper 5, 5612 AE Eindhoven, The Netherlands; m.soleimani@tue.nl (M.S.); r.r.m.joosten@tue.nl (R.R.M.J.); l.s.hazendonk@tue.nl (L.S.v.H.); sai.maddala1913@gmail.com (S.P.M.); 2 Department of Mechanical Engineering, Polymer Technology, Materials Technology Institute, Eindhoven University of Technology, Groene Loper 15, 5612 AE Eindhoven, The Netherlands; s.j.j.v.d.broek@tue.nl (S.J.J.v.d.B.); L.C.A.v.Breemen@tue.nl (L.C.A.v.B.); 3 Shell Technology Center Amsterdam Grasweg 31, 1031 HW Amsterdam, The Netherlands; 4 Institute for Complex Molecular Systems, Eindhoven University of Technology, Groene Loper 5, 5612 AE Eindhoven, The Netherlands

**Keywords:** hierarchical materials, diatom frustule, in situ mechanical testing, electron tomography

## Abstract

Investigating and understanding the intrinsic material properties of biogenic materials, which have evolved over millions of years into admirable structures with difficult to mimic hierarchical levels, holds the potential of replacing trial-and-error-based materials optimization in our efforts to make synthetic materials of similarly advanced complexity and properties. An excellent example is biogenic silica which is found in the exoskeleton of unicellular photosynthetic algae termed diatoms. Because of the complex micro- and nanostructures found in their exoskeleton, determining the intrinsic mechanical properties of biosilica in diatoms has only partly been accomplished. Here, a general method is presented in which a combination of in situ deformation tests inside an SEM with a realistic 3D model of the frustule of diatom *Craspedostauros* sp. (*C.* sp.) obtained by electron tomography, alongside finite element method (FEM) simulations, enables quantification of the Young’s modulus (E = 2.3 ± 0.1 GPa) of this biogenic hierarchical silica. The workflow presented can be readily extended to other diatom species, biominerals, or even synthetic hierarchical materials.

## 1. Introduction

Biocomposites such as shell, bone, and teeth have been an inspirational class of materials for designing new synthetic materials with tunable and advanced properties through biomimetic and bioinspired approaches [1,2,3,4,5]. The hierarchical morphology of such materials imparts extraordinary mechanical properties, achievable neither by their individual constituents nor any synthetic counterparts [6,7]. Thus, our ability to measure the intrinsic material properties of such complex biomaterials is an indispensable first step towards overcoming trial-and-error-based materials optimization in our synthetic efforts to design them with similarly advanced property levels for desired applications [8,9]. One such hierarchical biocomposite is the siliceous exoskeleton of diatoms, the frustule, consisting of two valves and several overlapping girdle bands [10,11,12]. Diatoms are unicellular photosynthetic algae that live in aquatic and terrestrial environments with a size range from 2 to 2000 µm [13]. The frustule with species-specific morphology [14] is a composite or hybrid material owing to the presence of various organic macromolecules such as silaffines and long-chain polyamines within its hydrated amorphous biogenic silica structure [15,16]. Because of their highly ordered micro- and nanostructures, diatom frustules have been used for various applications such as biosensors [17], microfabrication [18], separation applications [19], and for drug delivery [20]. In addition, the flexibility in the frustule formation provides an opportunity to incorporate different nonsessional metal ions into the frustules, inducing new characteristics such as photocatalytic activities upon Ti incorporation [21], electroluminescence and photoluminescence properties by Ge incorporation [22], and catalytic activities via Al addition [23], which are difficult to achieve by synthetic methods. The current understanding is that these hybrid organic–inorganic structures, i.e., the frustule, act as mechanical protection against diatom predators [24,25].

In recent years, researchers have shown an increased interest in the mechanical properties of frustules of a variety of diatom species by using several experimental techniques. For instance, mechanical properties of diatoms *Coscinodiscus* sp. and *Navicila pilliculosa* have been measured by AFM at different regions of their frustules, suggesting the key role of submicron morphological characteristics [26,27]. Conventional nanoindentation in combination with simulations studies has been performed on large diatoms such as *Coscinodiscus* sp. and *Synedra* sp., suggesting that the Young’s modulus and hardness of biosilica is species-specific [28]. Furthermore, in situ mechanical testing such as indentation on the whole diatom frustule or bending test on frustule’s fragments have been performed inside SEM to determine contact stiffness and Young’s modulus, respectively [29,30]. Besides these experimental studies, several simulations have also been conducted on simplified frustule models to obtain their mechanical properties [31,32,33]. For most of these computational studies SEM images of the frustules have been employed as initial data and the 3D models have been built based on them. Despite bringing new insights on the correlation between morphological and mechanical properties of the frustule, the simplified models used in these studies cannot represent the actual morphological features of frustules.

An appealing approach is to use current 3D imaging methods, i.e., tomography techniques [34,35,36,37], to obtain a realistic 3D model of the frustule. Surprisingly, only one 3D frustule structure of a large diatom *Didymosphenia geminata* (about 90 µm in length) has so far been acquired by nano X-ray computed tomography [38,39]. Subsequently, the obtained model has been used for FEM simulations in correlation with experimental results from in situ microindentation on the frustule to derive its mechanical properties [40]. Owing to the small size of most diatoms (about 60% of diatoms have a size range of 5–100 µm) [41] nano X-ray computed tomography can only be suited for characterizing few species and nanostructured features of most diatom frustules may not be identified due to the low spatial resolution (down to 400 nm) [42]. An alternative approach that provides nanometer resolution is electron tomography (ET) [43,44,45]. Previous study has established that via electron tomography, various morphological features such as pore size in the range of 10 nm as well as thickness of specimens can be determined quantitatively [36]. Thus, this technique may serve as versatile procedure to obtain realistic 3D morphologies of small diatom frustules with sufficiently high resolution.

Recently, there has been an increasing interest in ET investigations on different aspects of internal structures of diatom cells. For instance, by using cryo-ET, structure and spatial segregation of photosystem II complexes in thylakoid membranes of diatom *Phaeodactylum tricornutum* as well as photosystems II and I in the thylakoid membrane of diatom *Thalassiosira pseudonana* have been elucidated, respectively [46,47]. Furthermore, cryo-ET has been used to reveal the silicification process of diatom *Chaetoceros tenuissimu*, leading to a new observation regarding this process, where silicification is controlled by the cell machinery outside of the silica deposition vesicles (SDVs) [48]. However, to the best of our knowledge, no previous study has investigated diatom frustules by ET in the context of understanding the intrinsic mechanical properties of biosilica.

In this manuscript, we present a general approach to quantify the Young’s modulus of hierarchical biosilica on the example of diatom *Craspedostauros* sp. (*C.* sp.) at and below micrometer length scale by a combination of in situ mechanical testing inside SEM with a 3D model obtained from ET alongside FEM simulations. In addition, in situ manipulation of the valves is conducted, repositioning them in the best possible orientation for in situ mechanical deformation testing. The methodology presented enables the quantitative mechanical characterization of complex 3D morphologies, for instance, as found in hierarchical biocomposites.

## 2. Materials and Methods

### 2.1. Diatom Culture

*Craspedostauros* sp. (UTEX B679) was obtained from the UTEX Culture Collection of Algae, Austin, TX, USA. The cells of *C.* sp. were cultivated in artificial seawater supplemented with f/2 medium in a climate cabinet (Flohr, Utrecht, The Netherlands) under the following parameters: day/night cycle of 14/10 h, constant temperature of 23 °C, and a light intensity of 3000 Lux. In order to assure proper mixing of the growth medium, the cell culture flasks were shaken manually once per day during the entire experiment.

### 2.2. Sample Preparation for SEM Imaging and Mechanical Testing

The living cells were collected by centrifugation at 2600 rpm for 5 min (Minispin Centrifuge, Eppendorf, Hamburg, Germany). Then, the pellet was suspended in Mill-Q water and washed at least three times together with centrifugation to completely remove the salt and unreacted chemicals. The brownish-yellow pellet at the bottom centrifuge tube was dispersed in ethanol. After at least 10 times washing with ethanol, an off-white pellet was obtained and suspended in anhydrous ethanol. The resultant suspension was dried by a critical point dryer (Leica CPD 300 instrument, Wetzlar, Germany) to obtain intact diatom frustules (air drying of entire frustule often led to their structural collapse) [49]. This mild extraction process is intended to avoid any chemical or physical changes to the biosilica. In order to separate the valves from the girdle bands, the resultant suspension was centrifuged at a speed of 14,000 rpm and then sonicated by using a bath sonicator at room temperature (Bransonic ultrasonic cleaner, model 1510E-DTH, 42 kHz, Danbury, CT, USA) for about 5 min.

### 2.3. Scanning Electron Microscopy (SEM) and Energy Dispersive X-ray Spectroscopy (EDS)

For SEM imaging and EDS elemental mapping of the intact frustule of *C.* sp., a tiny amount of the critical point dried white pellet was deposited on a standard aluminum SEM stub. These experiments were conducted using a SEM Quanta 3D FEG instrument equipped with EDAX EDS detector (Thermo Fisher Scientific, previously FEI, Eindhoven, The Netherlands), at an acceleration voltage of 10 kV.

### 2.4. Mechanical Manipulation and In Situ Deformation Tests

To investigate the mechanical properties of isolated valves, 50 µL of the obtained suspension of the sonicated pellet (which separates valves from girdle bands and avoids structural collapse upon drying) was dropped on a silicon wafer attached to the SEM stub followed by drying in air. A micromanipulator (MM3A-EM, Kleindiek NanotechnikGmbH, Reutlingen, Germany) was used to reposition the valves (if necessary). The micromanipulator is also known for having high positional accuracy of about 10 nm for the mechanical movement in three axes inside the SEM chamber [50]. For manipulation of the valves, a tungsten probe was used with approximately 150 nm in radius mounted on the micromanipulator. Additionally, in situ deformation tests were conducted inside the SEM chamber using a highly sensitive (10 nN resolution) AFM-based force measurement sensor referred to as FMT-120 tip (Kleindiek NanotechnikGmbH, Reutlingen, Germany), which was mounted on the micromanipulator. Throughout the deformation testing, force–time data are provided by the force measurement system (FMS) which in correlation with SEM images can be transformed into force–displacement data [51]. It is worth nothing that due to limited rotational movement of the micromanipulator, all of the deformation tests were carried out while the sample holder and FMT-120 tip were aligned on their sides. In addition, owing to the anisotropic structure of the isolated valve and challenges related to positioning of large probes, flat punches which are commonly used for spherical specimens or biomaterial scaffold-like structures could not be employed in this study [52,53].

### 2.5. Scanning Transmission Electron Microscopy (STEM) Tomography and Segmentation

STEM imaging was carried out on the cryoTITAN (Thermo Fisher Scientific, previously FEI, Eindhoven, NB, The Netherlands) equipped with a field emission gun (FEG) and a high-angle annular dark field (HAADF) STEM detector (Fishione, Export, PA, USA). The microscope was operated at 300 kV acceleration voltage at an extraction voltage of 4500 V in microprobe STEM mode at a nominal magnification of 6.600× with frame time of 40 s, dwell time of 2 µs, and camera length of 1.150 m. The tomographic tilt-series was acquired over an angular range from −63° to 63°, at 2° increments. The raw data were aligned and reconstructed using IMOD [54]. Alignment was performed through manual tracking of fiducial gold markers throughout the entire tilt-series, followed by model fitting and minimization of the residuals. Subsequently, by using the simultaneous iterative reconstructive technique (SIRT) with 20 iterations, the tomogram was reconstructed. To segment and visualize the reconstruction, Avizo software (Avizo Fire 9.2, Thermo Fisher Scientific, Hillsboro, OR, USA) was employed, resulting in a surface mesh. It should be noted that for simplicity, small pores within the areola are not included in the segmentation.

### 2.6. FEM Simulations

Finite element method (FEM) simulations were conducted using the commercial software package MSC.Marc (MSC software corporation, version 14.0, California, CA, USA) in order to determine the intrinsic material properties of the biosilica of *C.* sp., i.e., its Young’s modulus. The mesh of the valve was generated based on the surface mesh that was experimentally obtained by electron tomography and consists out of approximately 450,000 s order tetrahedral elements. The material properties were assumed to be linearly elastic. The indenter was modeled as an impenetrable surface with an infinite stiffness. The valve was fixed in all three directions at one single bottom node in order to prevent rigid body modes and was at this same node supported by an impenetrable surface that acts as the sample support. To prevent rotations of the diatom in the x–y plane and y–z plane, two nodes, each located at the outer apexes of the valve, were constrained with respect to each other, and to prevent rotations in the x–z plane, one single node just below the indenter was constrained. Finally, a load case was specified that describes the z-displacement of the indenter identical to the compression experiment as performed inside the SEM. The average error value was calculated by determining the absolute force difference between the experimental and numerical curves for three valves, which was 8.5%.

## 3. Results and Discussion

### 3.1. Morphology and Elemental Composition of the Frustule of Diatom C.sp.

**Figure 1:**
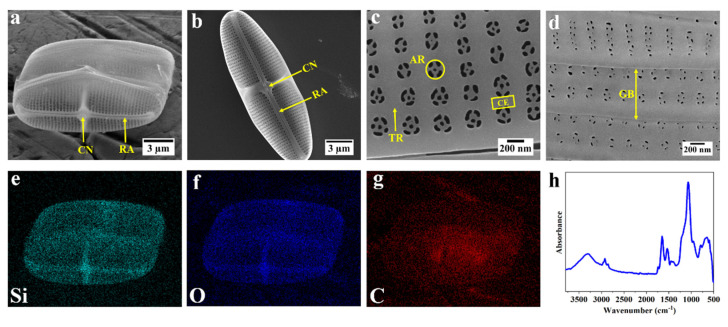
Morphology and elemental composition of the frustule of diatom *C.* sp. (**a**–**d**) SEM images of frustule of *C.* sp. (**a**) intact frustule of *C.* sp.; (**b**) an isolated valve deposited on its concave orientation showing the interior surface of the valve (RA = raphe; CN = central nodule); (**c**) porous area of the valve (TR = transapical rib; CE = cross extension; AR = areola); (**d**) overlapping girdle bands (GB); (**e**–**g**) EDS elemental mapping of the intact frustule; green = silicon, blue = oxygen, red = carbon; (**h**) FTIR spectra of frustule of *C.* sp.

*C.* sp. is a raphid pennate marine diatom. It has an imperfect rectangular prism shape, consisting of two valves and a number of girdle bands that connect them. Figure 1a shows an intact frustule of *C.* sp. lying on its girdle bands where the valve external face can be clearly seen. The valve comprises the most intricate structure of the frustule with various features such as a thick structure in the middle called central nodule, raphe ribs which are elongated throughout the valve, and several porous and nonporous regions (Figure 1b,c). As shown in Figure 1c, within the porous region there are several well-organized arrays of pores known as areolae (about 200 nm in diameter in a slight gradient, larger near the central nodule and smaller near the edge) that are connected by transapical ribs. Unlike the valves, the morphology of the girdle bands is much simpler and comprises some porous and nonporous regions (Figure 1d). In order to determine the elemental composition of the frustule, SEM–EDS elemental mapping was performed on the intact frustule, which is shown in Figure 1a. As can be seen in Figure 1e–g, a homogenous distribution of silicon, oxygen, and carbon are observed throughout the frustule. (Differences in signal intensity can be attributed to differences in local frustule thickness and not concentration variations.) Signals of Si and O are notably more pronounced than C within the frustule, specifically in the central nodule and raphe. (The strong C signal in the middle of the frustule is due to some residue of internal organelles being left behind on account of the mild washing procedure.) Fourier transformed infrared spectroscopy (FTIR) was employed to obtain some information about the chemical structure of the frustule. Figure 1h shows the typical characteristic peaks of diatom frustule, such as peaks at 948 and 1072 cm^−1^, which are assigned to the stretching vibration of silanol groups (Si–OH) and Si–O–Si bonds, respectively. In addition, several peaks in the range 1465 to 1750 cm^−1^ indicate the presence of various organic bonds such as N–H and C=O, while peaks at about 2854 and 2924 cm^−1^ represent the C–H stretching. Furthermore, an additional peak at about 3340 cm^−1^ shows the stretching of O–H groups [55,56,57].

### 3.2. Positioning of the Frustule for In Situ Mechanical Testing

**Figure 2:**
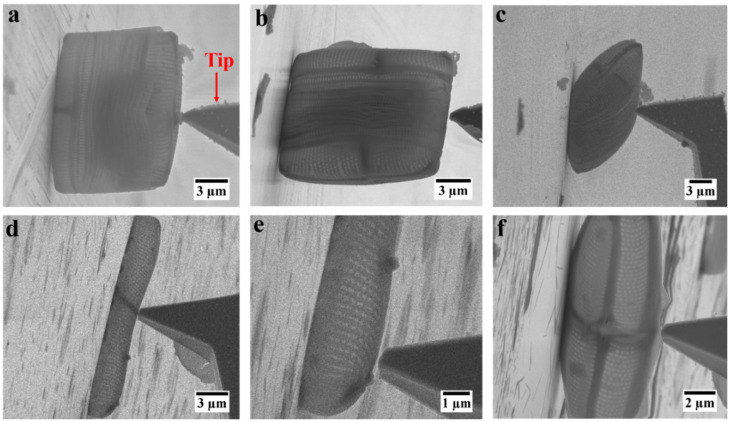
SEM images of different orientations of intact frustule and isolated valves of *C.* sp. for in situ deformation tests: (**a**) intact frustule standing upright; (**b**) intact frustule standing on girdle bands; (**c**) intact frustule laying on the girdle bands; (**d**,**e**) deformation test at different locations of an isolated valve laying flat; (**f**) deformation test on isolated valve standing on its side.

The first step in the procedure to measure the mechanical properties of the frustule of *C.* sp. was to establish the best possible positioning of the frustule prior to the mechanical testing. There were multiple options: first, conducting in situ mechanical probing on an intact frustule of *C.* sp. either on its valve or on its girdle bands, with different positioning. After deposition of several intact frustules on the SEM sample support, we found that in individual cases the frustule could stand up, on its valve or girdle band, as well as lay down, on its girdle bands (Figure 2a–c). However, as expected due their shape anisotropy, most of the frustules were laying down on their girdle bands. Attempted deformation tests with an indenter tip in the middle of a valve with the frustule standing up led to the movement of the entire frustule rather than deformation owing to the curved structure of the valve (Figure 2a). In addition, performing this experiment on the girdle bands regardless of their positioning led to the penetration of the tip into the frustule (Figure 2b,c). Most importantly, the size of the intact frustule made it challenging if not impossible to perform ET experiments. Therefore, in a somewhat simplified version, an isolated valve was chosen for such an experiment. Nonetheless, after deposition of separated valves and girdle bands on the sample support, most of the valves were laying down flat on the support (Figure 2d,e). In this position, due to the small distance between the curved part of the valve and the mounting surface, the potential effect of mounting surface on the acquired force–distance data, in case of touch down, was unknown. In addition, the applied force by the FMT-120 tip in this position was acting relatively locally, rather than deforming most of the structure of the valve. Thus, the only meaningful position was found where the valve was standing up on one side (Figure 2f). In this orientation, the valve could be deformed with minimal effects of the mounting surface.

**Figure 3:**
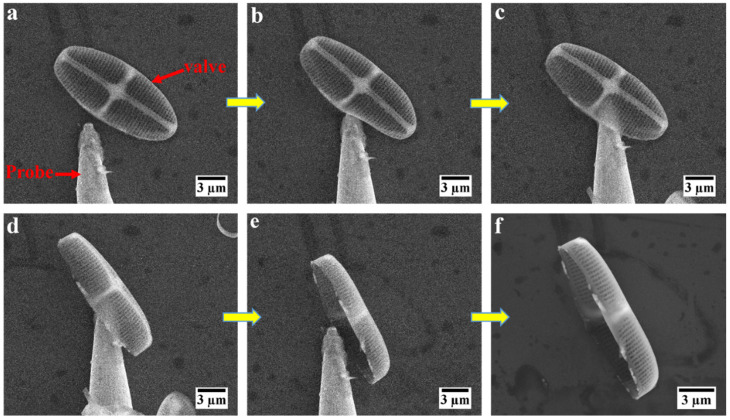
Mechanical manipulation of an isolated valve of diatom *C.* sp. (**a**–**f**) SEM image sequence acquired during repositioning of an isolated valve from its flat orientation to standing on its side.

However, the likelihood of finding this orientation after random liquid mediated deposition was low. Therefore, after deposition of the valve on the mounting surface, repositioning was needed prior to mechanical testing. To this end, a sharp tungsten tip mounted on the micromanipulator was employed to reposition valves to stand up on their sides. Figure 3a–f presents a sequence of the mechanical repositioning of an isolated valve requiring lifting, moving, and longitudinally rotating it to stand up on its side. This manipulation was conducted through mechanical manipulation alone without using any adhesive due to the possible alteration to the chemical and mechanical properties of the valves (Movie S1). In recent years, this type of in situ manipulation of small objects inside SEM has received attention due to its flexibility to clean the surface of 2D samples and transfer submicron specimens with high positional accuracy [58,59]. Similarly, here, we showed that mechanical manipulation can be used to reposition the specimens in which mechanical testing can certainly be carried out in a meaningful way.

### 3.3. In Situ Deformation Experiment, STEM Electron Tomography, and FEM Simulations

**Figure 4:**
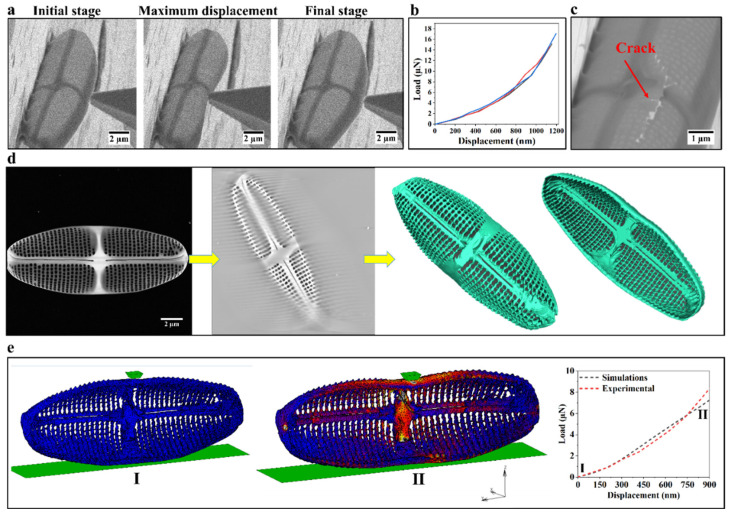
Overall workflow to quantify Young’s modulus of biosilica in the valve: (**a**) SEM images of an isolated valve at different stages during the in situ deformation test; (**b**) load–displacement curves for three valves obtained from one culture; (**c**) crack formed by deformation of the valve; (**d**) from left to right: STEM image of the valve, a slice of the reconstructed 3D intensity map of the valve, and 3D surface views of the valve; (**e**) initial undeformed (I) and final deformed (II) stages of the realistic valve model in FEM simulations and a comparison between experimental and simulated load–displacement curves.

To quantify the mechanical properties of the valve, a series of in situ indentor tip deformation tests were carried out with a force measurement sensor FMT-120. The FMT-120 tip was mounted on a micromanipulator for moving it into the valves to obtain force–time data which in correlation with the SEM images acquired constantly throughout the experiment translate into a force–displacement curve. Figure 4a shows different stages of a valve during this indentor deformation test. As can be seen, the valve exhibited a fully elastic behavior upon deformation and tip retraction without noticeable irreversible deformation (Movie S2). In accordance with previous studies, this elastic behavior has already been observed for other diatom species such as *Thalassiosira pseudonana* and *T. punctigera* [24,49]. Figure 4b presents the load–displacement curves of such experiments for three different valves of *C.* sp. measured up to a displacement of approximately 1100 nm. After the first cycle of deformation test, the displacement was increased to approximately 2500 nm, leading to fracture of the valve. It can be seen in Figure 4c that fracture took place in the middle of the valve under the region where force was applied by the FMT-120 tip. The cracks propagated along the central nodule and extended through the areola and transapical ribs.

Based on the experimental load–displacement data alone it is not possible to determine the intrinsic mechanical properties such as Young’s modulus of the biosilica of the valve because of its complex morphology. Therefore, ET was necessary to obtain a realistic model of the valve for detailed FEM simulations. ET was performed in microprobe STEM mode over an angular range from −63° to 63° at 2° increments (Figure 4d). Details of alignment, reconstruction, segmentation, and visualization (surface meshing compatible with FEM) are provided in the Materials and Methods section. In Figure 4d, a STEM image, a numerical cross-section through the reconstruction, and a 3D surface view of the valve containing all essential features (valve raphe, central nodule, and areole) are shown. For simplicity, the small pores within the areola were not included in the surface mesh. The mesh surface obtained and the experimental load–displacement data were utilized in the FEM simulations to determine the Young’s modulus of the biosilica constituting the valve. Figure 4e shows the undeformed and the deformed model in FEM alongside the comparison between the experimental and simulation load–displacement curves in the low regime of the curves. As shown in Figure 4e, the experimental results match the numerical data. Based on these findings, a Young’s modulus of 2.3 ± 0.1 GPa was determined for this biosilica. In accordance with the present results, previous studies have suggested that Young’s modulus of diatom biosilica can vary from 0.347 to 300 GPa [26,60]. Despite the fact that the value obtained here is significantly lower than the reported average Young’s modulus of fused silica [61], it must be noted that the frustule is a biocomposite and therefore exhibits considerable variation in mechanical properties compared to fused silica. Although in the present study only one diatom species with a limited number of samples was studied, the results obtained agree with the previous investigation, providing further support for the hypothesis that the mechanical properties of diatom frustules are also species-specific, in addition to their morphological features [28].

## 4. Conclusions

A versatile approach by a combination of in situ deformation, electron tomography, and FEM simulations was developed to determine the mechanical properties of the diatom frustule. The micromechanical performance of the valves of diatom *C.* sp. was studied by in situ deformation tests inside an SEM. ET was carried out to obtain a realistic 3D model of the complex valve morphology including all essential features. Based on the load–displacement data obtained and the 3D surface mesh of the valve, FEM simulation led to determine Young’s modulus of this biosilica. The workflow presented can be readily extended to other diatom species, biominerals, or even synthetic hierarchical materials.

## Data Availability

The datasets generated during and/or analyzed during the current study are available from the corresponding authors upon reasonable request.

## Supplementary Materials

The following supporting information can be downloaded at: https://www.mdpi.com/article/10.3390/nano12091549/s1, Movie S1: In situ manipulation of an isolated valve, Movie S2: In situ deformation test on an isolated valve.
